# Comparative performance of activated sludge and waste stabilization ponds for the removal of pollutants and pathogens in full-scale wastewater treatment plants in Egypt

**DOI:** 10.1038/s41598-026-35933-4

**Published:** 2026-02-05

**Authors:** Marwa A. Kamel, Neveen Magdy Rizk, Mahmoud Gad, Gamal K. Hassan, Aly Al-Sayed, Mariam E. Fawzy, Mohamed Azab El-Liethy, Ibrahim Ahmed Hamza

**Affiliations:** 1 Environmental Virology Lab., Water Pollution Research Department, Environment and Climate Change Research Institute, National Research Centre, 33 El- Buhouth, Giza, 12622 Egypt; 2 Water Pollution Research Department, Environment and Climate Change Research Institute, National Research Centre, 33 El- Buhouth, Giza, 12622 Egypt; 3 Water Pollution Research Department, Environment and Climate Change Research Institute, National Research Centre, 33 El- Buhouth, Giza, 12622 Egypt; 4 Environmental Microbiology Lab., Water Pollution Research Department, Environment and Climate Change Research Institute, National Research Centre, 33 El- Buhouth, Giza, 12622 Egypt

**Keywords:** Activated sludge, Waste stabilization ponds, Physicochemical parameters, Microbial indicators, Bacteria, Viruses, Environmental sciences, Microbiology, Water resources

## Abstract

The discharge of inadequately treated wastewater poses serious public health and environmental risks worldwide. In this study, the performance of two wastewater treatment plants (WWTPs) was evaluated for their ability to remove pathogenic viruses and bacteria, organic and nitrogen compounds, bacteriophages, and bacterial indicators. WWTP-A employs activated sludge treatment technology, whereas WWTP-B utilizes waste stabilization ponds (WSP). The removal rates of chemical oxygen demand (COD) and biological oxygen demand (BOD) were higher in WWTP-A than in WWTP-B, indicating the limited efficiency of the WSP system in treating heavily contaminated sewage. In WWTP-A, bacterial removal rates ranged between 3.9log_10_ for *Listeria* spp. and 6.2 log_10_ for *Escherichia coli* (*E. coli*), while in WWTP-B, removal ranged from 3.2 log_10_ for *Pseudomonas aeruginosa* to 5.3 log_10_ Fecal Streptococci*.* The viral reduction rate by WWTP-A ranged from 0.9 log_10_ for crAssphage to 3 log_10_ for human adenovirus (HAdV), whereas in WWTP-B, viral removal ranged between 0.8 log_10_ for somatic coliphages (SOMCPH) and 3 log_10_ for crAssphage. CrAssphage exhibited significant influent–effluent reduction in both WWTP-A and WWTP-B (Wilcoxon test, p < 0.05). In contrast, significant HAdV reduction was observed only in WWTP-A, SOMCPH only in WWTP-B, while RoV showed no significant reduction in either system. These findings highlight limitations in the ability of conventional treatment processes to consistently remove viral contaminants from treated effluent and underscore the need for improved management strategies to enhance effluent quality.

**Supplementary Information:**

The online version contains supplementary material available at 10.1038/s41598-026-35933-4.

## Introduction

As global water demand continues to rise while resources remain limited and are increasingly affected by climate change, the need for wastewater reuse has become inevitable. According to the World Health Organization (WHO), over half of the world’s population lives in water-stressed areas, with access to contaminated water from sewage and septic tanks^1^. FAO water accounting reports in 2020 stated that Egypt is among the countries that are at risk of food insecurity due to water shortage, based on its location in closed river basins and limited options for expanding water resources^2^.

Wastewater reuse has been recognized recently as a mean of water sustainability and meeting the United Nations Sustainable Development Goals (SDGs). To address freshwater scarcity, treated wastewater is increasingly utilized for various purposes, particularly in agriculture^3–5^. However, the reuse or discharge of treated sewage poses significant health risks, as it contains a diverse range of human pathogens. According to the WHO, waterborne diseases are responsible for approximately 505,000 diarrheal deaths annually, particularly in low- and middle-income countries^6^.

Generally, wastewater reuse standards specify chemical and microbiological quality criteria. In Egypt, the ministerial Decree 48/1982 and Code 501/2015 limit microbiological monitoring to indicator organisms, mainly total coliforms (≤5×10^3^ MPN/100 mL) and nematode ova (<1 egg/L), which are used as surrogates of fecal contamination (Table S1)^7^. However, specific viral, protozoal and bacterial pathogens are not explicitly regulated as effluent quality targets. Many enteric viruses, including HAdV and RoV, are more resistant than these bacterial indicators to conventional wastewater treatment, so residual viral contamination may persist even when indicator-based standards are met^8^. The absence of explicit viral monitoring requirements in many wastewater reuse regulations can therefore increase the risk of virus transmission, particularly where treated wastewater is applied in agriculture. Potential exposure routes include consumption of contaminated produce, dermal contact with reclaimed water, and inhalation of aerosolized viral particles during irrigation, and even low viral doses may infect vulnerable groups such as children and immunocompromised individuals^9^. Accordingly, incorporating viral detection and control measures into wastewater reuse guidelines is essential to reduce waterborne infection risks.

Different wastewater treatment technologies are used across Egypt’s 412 WWTPs, mainly activated sludge and WSP systems^10^. WSPs combine algal and bacterial treatments, whereas activated sludge systems rely solely on bacteria^11,12^ . The former are common in small towns due to their low cost and simple operation. In contrast, activated sludge systems are used in larger cities due to their higher and more consistent efficiency^13,14^ . The performance of WSPs depends on environmental conditions, such as sunlight, temperature, and evaporation rates, and they require longer hydraulic retention times (HRTs). Activated sludge systems, although more reliable, are more costly and require regular maintenance. A comparative assessment of both systems remains essential for optimizing wastewater management in Egypt. Despite the global prevalence of activated sludge, many developing countries still rely on WSPs due to their affordability and ease of operation compared to advanced wastewater treatment technologies^15–17^. However, inadequate wastewater treatment increases the risk of transmitting infectious disease, either by discharging a large number of pathogens into the environment or through contaminating drinking, surface, and groundwater^18^.

In this context, the primary objective of this study was to evaluate the removal efficiency of chemical and microbiological contaminants—including bacterial indicators, human pathogenic viruses, and bacteriophages—in two full-scale wastewater treatment plants (WWTPs) in Egypt operating under comparable climatic and influent conditions: one based on an activated sludge system (WWTP-A) and the other on waste stabilization ponds (WWTP-B). While previous studies in Egypt and the region have reported microbiological or physicochemical performance of individual treatment systems, they are primarily limited to single technologies, laboratory- or pilot-scale investigations, or short-term monitoring focusing on either bacterial indicators or selected pathogens^12,19–21^.

To the best of our knowledge, this study provides the first integrated, long-term (seven-month), side-by-side field comparison of activated sludge and WSP technologies at full operational scale in Egypt, combining conventional water quality parameters with the concurrent detection of bacterial pathogens, human enteric viruses, and bacteriophages. By generating comprehensive, technology-specific performance data under real operational conditions in a water-scarce MENA setting, this work delivers novel, region-specific evidence that directly supports risk-based wastewater reuse strategies and informs technology selection to meet regulatory requirements while reducing environmental and public health risks.

## Materials and methods

### Wastewater treatment plants site and sampling

A total of 28 wastewater samples were collected monthly from the influent and effluent of El-Gabal El-Asfar (WWTP-A) and Atfih (WWTP-B) over a period of seven months. Samples were collected in sterile 1L glass bottles, transported in an icebox within 2 h to the National Research Centre laboratories, and analyzed on the same day according to APHA standard methods^22^.

WWTP-A, the largest wastewater treatment facility in Egypt, is located in El-Qalyubia Governorate. It operates a secondary treatment system based on activated sludge technology, with a flow rate of 2,500,000 m^3^/day and serving approximately 12 million inhabitants. The treated effluent is reused for irrigation on 150,000 acres of landscaping, reducing dependence on non-renewable water sources.

While WWTP-B is located in the Atfih rural region, approximately 55 km south of Giza Governorate, west of the Nile River, and within Giza city, this wastewater treatment facility has a capacity of 45000 m^3^/day. The system consists of three sequential treatment stages: an anaerobic pond, a facultative pond, and a maturation pond. This plant is designed to comply with Egyptian legislation for the reuse of municipal wastewater in agriculture, serving an estimated population of 368,693 inhabitants. A table summarizing the wastewater reuse standards in Egypt^7^ has been added to the Supplementary Materials (Table S1).

### Physicochemical analytical methods

The influent and effluent wastewater samples were analyzed according to APHA^22^. In situ measurements for pH, temperature, total dissolved solids (TDS), and electric conductivity (EC) were recorded using a multi-parameter (HI 98195) portable meter. The chemical analysis includes: chemical oxygen demand (COD), biological oxygen demand (BOD), total phosphorus (TP), ammonia-nitrogen (NH_4_-N), nitrite (NO_2_), nitrate (NO_3,_), total Kjeldahl nitrogen (TKN) and total nitrogen (TN) were measured according to^22^.

### Bacteriological examination

Bacterial indicators, including total coliforms (TC), fecal coliforms (FC), *E. coli* (EC) and fecal streptococci (FS) were determined in this study. The Most Probable Number (MPN) method was used to determine the bacterial indicator according to APHA^22^. TC was cultured in Lauryl Tryptose broth and confirmed using Brilliant Green Bile Lactose Broth, while FC was cultured in EC broth and incubated at 44.5°C for 24 hours. *E. coli* identification was confirmed by adding Kovac’s reagent to positive EC broth tubes. FS was determined using Azide Dextrose Broth, followed by sub-culturing on Pfizer selective agar. The results were expressed as MPN-index per 100 mL. All microbiological culture media were obtained from HiMedia Co., India.

In addition to bacterial indicators, selected pathogenic bacteria were investigated, including *Salmonella* spp., *Pseudomonas aeruginosa*, *Staphylococcus aureus*, and *Listeria* spp. Selective media were used for enumeration: *Salmonella* spp. on HiCrome Improved Salmonella Agar, *P. aeruginosa* on HiFluoro™ Pseudomonas Agar, *S. aureus* on HiCrome Staph Selective Agar, and *Listeria* spp. on HiCrome Listeria Agar using the spread plate technique according to APHA^22^. Bacterial counts were expressed in colony-forming units per 100 mL (CFU/100 mL). For bacterial identification, the Biolog GEN III system (Biolog Inc., USA) was employed. Fresh bacterial isolates were suspended in 10 mL of inoculating fluid A and dispensed into a microplate (100 μL per well), which was incubated at 37°C for 18–24 hours. Bacterial species were identified based on their metabolic profiles using the semi-automated Biolog system^23^.

### Viral detection

#### Virus concentration

A total of 100 mL of influent and 200-400 mL of effluent samples from WWTPs were concentrated using the virus adsorption-elution (VIRADEL) method^24^. Samples were centrifuged at 5000 rpm for 10 min to remove solid particles. The supernatant pH was adjusted to 3.5, then filtered through a nitrocellulose membrane filter with a 0.45µm pore size and a 47 mm diameter. Viruses were eluted from the membrane using elution buffer (3% beef extract, 0.05 M glycine, pH 9.5). The eluates were kept at -20 °C until nucleic acid extraction.

#### Detection of somatic coliphages

The quantification of somatic coliphage was carried out through the double agar layer plaque test, utilizing *E. coli* (ATCC 13706) as the bacterial host, in accordance with the standard method of the International Organization for Standardization, ISO 10705-2^25^.

#### Viral nucleic acid extraction

Nucleic acid was extracted from 200 μL of the virus concentrate obtained by the VIRADEL methods, using GenJet Viral RNA/DNA extraction kit (Thermo Scientific, USA) according to the manufacturer’s instructions. The extracted nucleic acid was kept at -20 °C until qPCR was performed.

#### Reverse transcription of human rotavirus (RT-PCR)

For reverse transcription reactions, 2.5 μL of extracted RNA was used as a template in a reaction mixture containing 0.5 μM of RoV A forward and reverse primer (RoV F, and RoV R), 2 μL of 10 mM DNTPs, 4 μL of 5x RT-Buffer, 1 μL of RT-enzyem (ReversrAid Reverse transcriptase, Thermo Scientific, USA) and nuclease-free water to a final volume of 20 μL were mixed and incubated for 60 min at 40 °C.

#### Quantification of viral genome by qPCR

The concentrations of RoVs, HAdV, and crAssphage were determined by qPCR to evaluate the performance of the two selected WWTPs. Table 1 lists all the primers used in the current study. All viruses were quantified using the TaqMan probe assay, except for somatic coliphages. TaqMan real-time qPCR reactions were conducted in a total volume of 20 μL, using 10 μL of GoTaq® Probe qPCR (Promega, Madison, USA), 0.5 μM for both forward and reverse primers, 0.2 μM Taqman probe, and 5 μL DNA or 2µl cDNA template. The qPCR procedure included an initial activation step of 95°C for 2 min for HotStart Taq DNA Polymerase, followed by 45 cycles of 2-step cycling for 15 s at 95 °C and 1 min at 60°C. To eliminate the possibility of cross-contamination, negative controls (NTC) containing nuclease-free water were incorporated in every run^26^. All NTCs consistently yielded negative results in the qPCRs. All PCR runs demonstrated efficiencies between 91–105% and R^2^ values between 0.92 and 0.98. The PCR inhibitory effect was investigated by diluting the purified nucleic acid extract prior to RT-PCR amplification, as previously documented^27^. The amplification and subsequent data analysis were conducted using the A CFX96 Touch™ Real-Time PCR Detection System (Bio-Rad).

**Table 1:** Nucleotide sequences of primers and probes used in q (RT) PCR assay.

Virus	Target gene	Primer name	Sequence (5`–3`)	Size (bp)	Reference
HAdV	Hexon	AQ1	GCC ACG GTG GGG TTT CTA AACTT	132	^28^
		AQ2	GCC CCA GTG GTC TTA CAT GCA CAT C		
		AdV-Probe	[6FAM] TGCACCAGACCCGGGCTCGGT ACTCCGA [BHQ1]		
RoV A	VP6	RoV F	ATGGATGTCCTGTACTCCTTGTCAAAA	128	^29^
		RoV R	TTCCTCCAGTTTGRAASTCATTTCC		
		Rota_P1	[6FAM] ATAATGTGCCTTCGACAAT-[MGBNFQ]		
		Rota_P2	[6FAM]AATATAATGTACCTTCAACAAT-[MGBNFQ]		
CPQ_56	orf00024	056F1	CAGAAGTACAAACTCCTAAAAAACGTAGAG	125	^30^
		056R1	GATGACCAATAAACAAGCCATTAGC		
		056P1	[FAM]AATAACGATTTACGTGATGTAAC [MGB]		

Human adenovirus (HAdV); group A rotavirus (RoV A); crAssphage (CPQ_56).

### Statistical analysis

Box plots were used to present microbiological data, comparing levels between WWTPs-A and B. The lines within each box indicate the medians, while the outer edges represent the 25th and 75th percentiles. Whiskers show min–max values. The Wilcoxon test was used for comparing microbial concentrations between the inlet and outlet samples within each WWTP, and the Mann–Whitney U test was applied for comparisons between WWTP-A and WWTP-B with P < 0.05 considered statistically significant. Principal component analysis (PCA) was used to explore multivariate patterns in physicochemical data and to compare samples from the two WWTPs. Principal component analysis (PCA) was used to explore multivariate patterns in physicochemical data and to compare samples from the two WWTPs.

## Results and discussion

### Characteristics of raw and effluent wastewater samples 

Discharging effluents that fail to meet regulatory limits for organic load (e.g., BOD, COD) and microbiological indicators can adversely impact the environment, contaminate receiving waters and increase public health risks. Therefore, evaluating the efficiency of contaminant removal by WWTPs is essential for assessing public health and environmental risks.

**Fig. 1:**
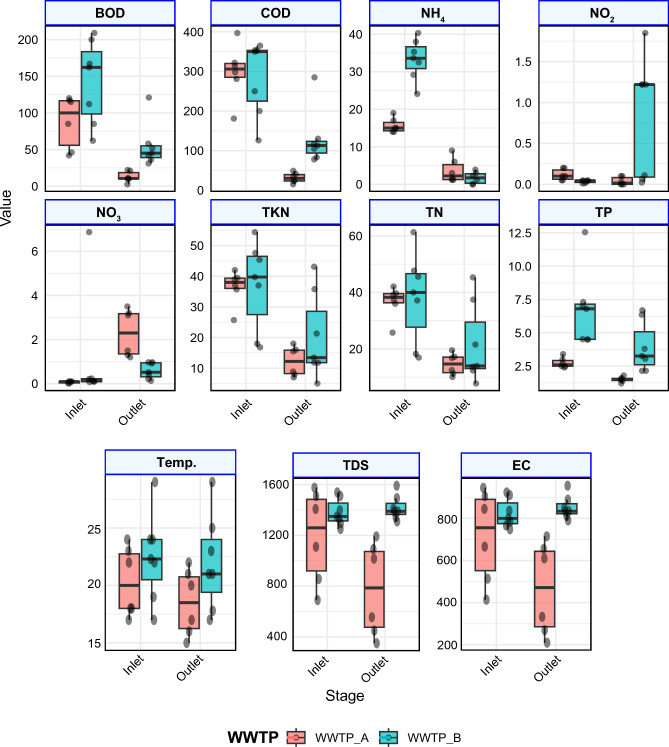
Physicochemical parameters including COD, BOD, TN, TP, Temp, TDS and EC values of WWTP-A and WWTP-B.

Figure 1 shows influent and effluent COD concentrations and removal efficiencies for WWTP-A. Influent COD ranged from 181 to 397 mg/L, indicating substantial variation in organic load, whereas effluent COD ranged from 15 to 49 mg/L, corresponding to a mean removal efficiency of 89.9%. The consistently low effluent COD demonstrates the high performance of the activated sludge system in removing biodegradable organic matter. For WWTP-B, influent COD ranged from 200 to 365 mg/L with relatively limited variation, while effluent COD varied widely between 78 and 285 mg/L, yielding an average removal efficiency of 56.3%. This lower and less stable COD removal indicates that, under the conditions of this study, the WSP system provided only limited treatment of the incoming organic load. This may be due to the presence of high concentrations of algae or phytoplankton in the final effluent, which contribute a substantial amount of particulate organic matter measured as COD. Sharafi et al.^31^ reported that COD removal efficiency was higher in the activated sludge WWTP compared to the WSP.

Fig. 1 shows the concentrations of BOD in WWTP-A and the removal efficiency for the treatment plant. The difference in BOD concentrations between the influent and effluent wastewater shows the efficiency of the treatment plant in removing organic compounds, as represented by the BOD parameter. The influent BOD of WWTP-A ranged from 42 mg/L to 120 mg/L. However, the effluent BOD ranged from 5 mg/L to 17 mg/L, with an average removal efficiency of 82.4%. The high BOD removal efficiency demonstrates the effectiveness of the WWTP, highlighting that the aerobic biological system is highly efficient in treating wastewater. Our results were in agreement with other reports, which stated that BOD removal efficiency for a typical activated sludge process ranges from 75-95% using the extended aeration process according to the inlet characteristics^32^.

In WWTP-A, the high COD removal efficiency appears somewhat counterintuitive given the relatively low BOD₅/COD ratio of the influent, which indicates a substantial fraction of slowly or non-biodegradable organic matter. This suggests that the observed COD reduction is not only due to biological oxidation, but also to multiple mechanisms that take place in the activated sludge process, such as adsorption, bioflocculation, and physical entrapment^15–17^. Extended solids retention time enhances contact between wastewater and sludge, promoting the adsorption of non-biodegradable organic matter onto sludge flocs, which are then removed during settling. All these mechanisms contribute to COD removal in the activated sludge system^15–17,33^.

The influent BOD for WWTP-B ranged from 85 mg/L to 209 mg/L, and the effluent ranged from 31 mg/L to 121 mg/L, with an average removal of 56%. The low removal of BOD indicated low efficiency of the WWTP, suggesting that the WSP system was less effective than the activated sludge system for treating sewage sludge. The low removal observed may reflect several factors, including suboptimal operation and maintenance of the WWTP and the presence of algae in the effluent. Moreover, decay and die-off of algal biomass produced under suboptimal operating conditions can contribute substantial biodegradable organic matter^34^, which is reflected in elevated BOD levels. This finding aligns with El-Gohary et al.^35^, who reported that the average BOD effluent from the WSP after sewage sludge treatment was 94 mg/L. These results highlight that aerobic bacteria in activated sludge, which continuously utilize oxygen to degrade organic matter, are more effective than the natural oxygen-dependent process in the WSP system^34^. Moreover, the characteristics of wastewater and the design of the WWTP may influence the removal efficiency of COD and BOD, potentially causing significant differences between the two investigated WWTPs.

Recent comparative studies have demonstrated significant differences in the removal of organic matter between wastewater treatment technologies. For activated sludge systems, Wang et al.^36^ documented COD removal efficiencies ranging from 80% to 99.25% and BOD removal efficiencies of 99% in aerobic processes. In contrast, WSPs achieve lower removal efficiencies; Mahapatra et al.^37^ reported BOD and COD removal rates of typically 70–94%, depending on the pond design and hydraulic retention time. The variations in the raw wastewater characteristics and treatment efficiencies of both systems may be attributed to fluctuations in the load and composition of the raw wastewater entering each treatment system. The considerable differences in treatment performance could also result from varying operational conditions, such as temperature and aeration, as well as the inherently dynamic nature of the two biological systems^38,39^.

Regarding TKN characteristics (Fig. 1), influent concentrations ranged from 25.7 to 42 mg/L in WWTP-A and from 18 to 54.4 mg/L in WWTP-B, indicating broadly similar nitrogen loads entering both systems. Average TKN removal efficiencies were 66.8% for WWTP-A and 61.6% for WWTP-B, with Wilcoxon signed-rank tests confirming that influent–effluent reductions were statistically significant (p < 0.05). Comparable TKN removal has been reported for WSP, such as in Mèze, France, where average removal efficiencies of 68% were attributed to favorable nitrification–denitrification conditions and active biomass development under appropriate operational conditions^40^.

With respect to TP characteristics, as shown in Fig. 1, the phosphorus in raw wastewater ranged from 2.5 mg/L to 3.8 mg/L for WWTP-A; however, these concentrations were higher in WWTP-B, ranging from 4.5 mg/L to 7.8 mg/L. The average removal efficiency for WWTP-A was 51.1%, whereas it was 33.9% for WWTP-B. Statistical analysis (Wilcoxon signed-rank test) confirmed that TP reductions in both WWTPs were significant (p < 0.05). This further supports that the activated sludge wastewater treatment plant was more effective than the WSP in removing phosphorus from sewage wastewater. Phosphorus removal in activated sludge systems takes place by assimilation, chemical precipitation and sorption mechanisms^41^. However, in WSP algal biomass utilizes phosphorus for growth and releases phosphorus in wastewater during die-off, the remaining phosphorus settles in sludge. Statistical analysis using the Wilcoxon signed-rank test for paired influent–effluent samples within each WWTP and the Mann–Whitney U test for comparisons between WWTP-A and WWTP-B confirmed that reductions in COD, BOD, TP, TKN, and TN were statistically significant (p < 0.05). WWTP-A consistently achieved greater reductions than WWTP-B, highlighting the superior efficiency of the activated sludge system and the comparatively lower and more variable performance of the WSP.

**Fig. 2.**
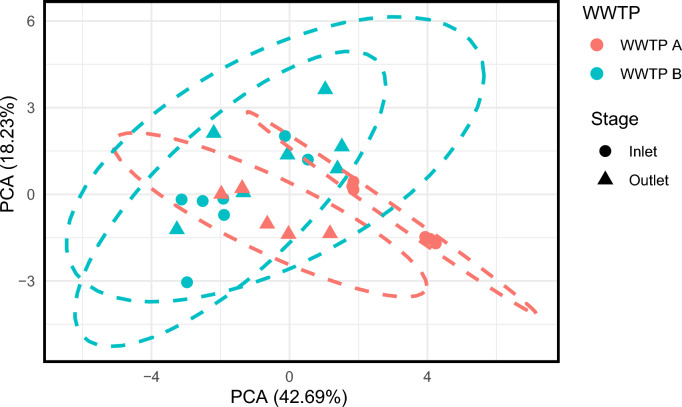
Principal component analysis (PCA) of physicochemical parameters in influent and effluent samples from WWTP A and WWTP B. Points show individual samples (color = WWTP, shape = stage), and the first two components explain 42.69% and 18.23% of the variance. Dashed ellipses indicate 95% confidence intervals for each WWTP, highlighting within-plant variability and the overall separation between influent and effluent.

Additionally, multivariate principal component analysis (PCA) of physicochemical parameters from influent and effluent samples in Fig. 2 revealed a clear separation between treatment stages along the first principal component, indicating that wastewater treatment imposes a consistent and dominant shift in water‑quality profiles. The partial overlap of clusters from WWTP‑A and WWTP‑B suggests that, despite their different process configurations, both plants receive influent of broadly similar composition and achieve comparable overall treatment performance. Additional drivers of variability may include differences in hydraulic retention time, sunlight exposure, and seasonal temperature variability, which are particularly relevant for WSP systems.

### Bacteriological parameters

Routine microbiological assessments of wastewater are crucial for determining water quality to prevent outbreaks^42^. Bacterial indicators such as TC, FC, *E. coli*, and FS are routinely used to assess water and wastewater treatment processes^43^. Therefore, in the present study, bacterial indicators, including TC, FC, E. coli, and FS, were determined at the inlet and outlet of WWTP-A & B.

**Fig. 3:**
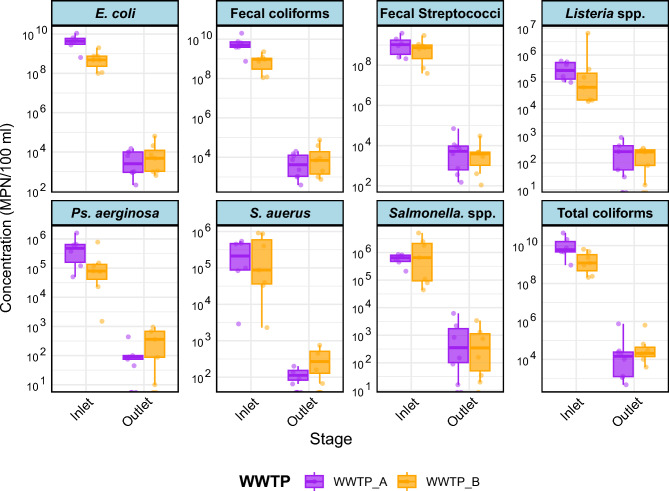
Box-whiskers plot with range (min-max) of bacterial concentrations in inlet and outlet samples of WWTP-A and WWTP-B.

At WWTP-A, influent concentrations of TC, FC, E. coli, and FS ranged from 9.15–10.15 log₁₀ MPN/100 mL and were reduced to 3.74–5.00 log₁₀ MPN/100 mL in the effluent (Fig. 3). These reductions reflect effective bacterial removal through flocculation, sedimentation, and biological treatment inherent to the activated sludge process. Similar log₁₀ reductions have been reported in other Egyptian activated sludge WWTPs^44,45^. By contrast, wastewater in developing countries typically exhibits higher bacterial loads than in developed regions^46^; for example, influent TC and E. coli in Hamburg, Germany were 3.56–5.90 and 3.40–5.64 log₁₀ CFU/100 mL, respectively^47^. The higher reductions observed in WWTP-A suggest that activated sludge systems are particularly effective under high influent loading, with flocculation and clarification providing consistent removal of both indicator bacteria and particulate-associated pathogens.

At WWTP-B, influent bacterial loads were slightly lower (8.82–9.34 log₁₀ MPN/100 mL) and effluent concentrations decreased to 3.82–5.04 log₁₀ MPN/100 mL (Fig. 3). The observed ~4–5 log₁₀ reductions are consistent with typical WSP performance in Egypt^12^ and are likely enhanced by the region’s high ambient temperatures and solar exposure, which can promote microbial inactivation. Mahapatra et al.^37^ reported 2–5 log reductions of bacterial indicators across temperature extremes (−2 to 40 °C), highlighting the role of sunlight-driven UV photobiological damage and seasonal temperature differences in WSP performance. While UV intensity and temperature were not measured in the present study, the arid climate, elevated ambient temperatures, and strong solar exposure likely contributed to the observed bacterial reductions. Comparison with WWTP-A indicates that despite WSPs benefiting from environmental factors, their removal efficiency remains more variable and generally lower than activated sludge systems. Nevertheless, effluent concentrations frequently exceeded reuse guideline limits, illustrating the inherent limitations of pond-based systems in consistently achieving microbiological safety without additional treatment barriers^48^.

Despite significant bacterial removal, pathogenic microorganisms may persist in treated effluents, posing potential public health risks^49^. Moreover, inadequately treated wastewater can disrupt natural microbial community functions^50^. Therefore, Salmonella spp., *P aeruginosa, S aureus*, and Listeria spp. were assessed in influent and effluent samples from both WWTPs (Fig. 3).

Pathogenic bacteria including Salmonella spp., *P aeruginosa, S aureus*, and Listeria spp.—were also monitored to evaluate potential health risks. At WWTP-A, influent levels ranged 5.43–5.78 log₁₀ CFU/100 mL and decreased to 2.30–3.04 log₁₀ CFU/100 mL in the effluent. Comparable reductions have been observed in other Egyptian treatment plants^51^ and globally, indicating that incomplete bacterial removal is a widespread challenge^52^. At WWTP-B, log₁₀ reductions for pathogens ranged 3.2–5.3, reflecting moderate removal efficiency. The data suggest that particulate-associated pathogens are removed more efficiently in activated sludge than in WSPs, emphasizing the importance of both treatment design and environmental drivers in pathogen reduction.

Overall, bacterial removal efficiency varied by indicator and treatment system, with greater reductions consistently observed in the activated sludge process. All reductions were statistically significant (Wilcoxon signed-rank test, p < 0.05). Despite substantial removal, treated effluents from both systems still contained considerable pathogen loads, suggesting potential risks to receiving waters if discharged or reused without additional protection. These findings underscore that bacterial removal is influenced by both technological factors (treatment type) and environmental conditions (temperature, sunlight), with activated sludge providing more reliable performance and WSPs showing variable efficiency depending on climatic drivers. The comparative analysis highlights where each system may succeed or fail in protecting public health.

### Abundance of viruses in wastewater

Evaluating the WWTP efficiency in removing viruses is necessary because the traditional wastewater treatment process may not always be efficient in eliminating these viruses^53–56^. Moreover, several studies have confirmed that bacterial indicators do not consistently correlate with the presence of enteric viruses or accurately reflect viral-associated health risks.

**Fig. 4:**
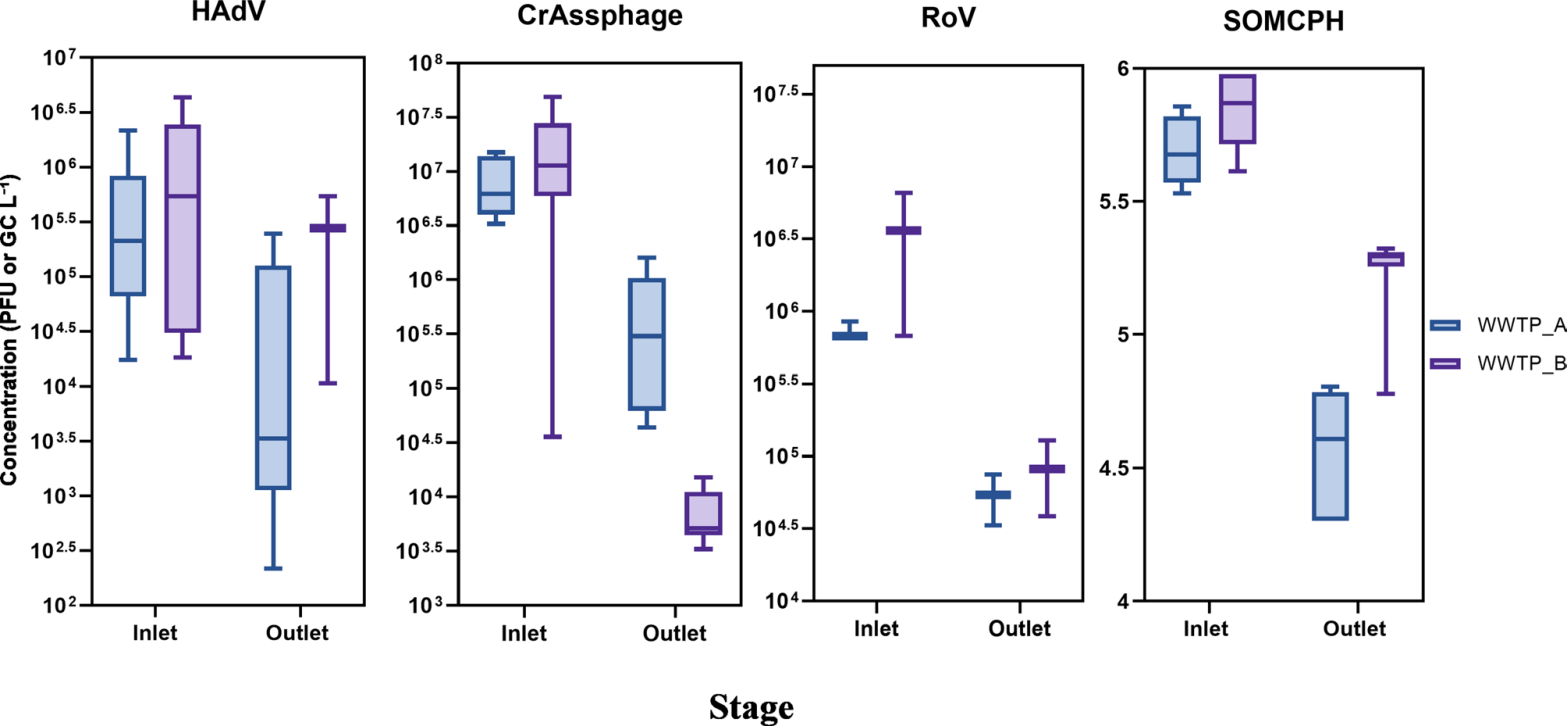
Box-whiskers plot (min-max) of median concentration of viruses (GC/l; PFU/l) in the inlet and outlet of WWTP-A and WWTP-B.

In this study, two prevalent enteric viruses in the aquatic environment, RoV A and HAdV, and two abundant bacteriophages in sewage, SOMCPH and crAssphages, were used to assess the viral removal in WWTPs. As shown in Fig. 4, crAssphage exhibited the highest concentrations in influent and effluent samples, followed by HAdV, RoV A, and SOMCPH. Viral reduction rates varied between ~0.8–3 log₁₀, with WWTP-A generally achieving greater reductions, particularly for HAdV. Statistically significant reductions were observed for crAssphage and HAdV (Wilcoxon test; p < 0.05), whereas RoV A and SOMCPH showed no significant changes, indicating differential removal efficiency among viral targets.

The superior bacterial removal observed in WWTP-A compared with WWTP-B likely reflects features of the conventional activated sludge process, including high biomass concentrations, floc formation promoting bacterial adsorption, and efficient clarification. In contrast, viral indicators and enteric viruses are smaller, often remaining freely suspended or associated with fine particles, making them less susceptible to physical removal and more dependent on specific inactivation mechanisms^57–59^.

In WWTP-B, long hydraulic retention times, high sunlight exposure, and elevated temperatures in stabilization ponds may enhance viral inactivation via photolysis, endogenous decay, and predation. However, high suspended solids and algal biomass can shield viruses from UV, limiting effective inactivation^60,61^. These operational and environmental factors explain the modest and variable viral reductions (~0.8–3 log₁₀) observed, particularly for more resistant non-enveloped viruses^62^.

Differential viral removal in WWTP-B—with greater reductions of crAssphage and SOMCPH than HAdV or RoV A—reflects combined effects of viral structure and environmental conditions. Kohn et al.^63^ demonstrated that viral structure determines photoinactivation susceptibility, while Schwarz et al.^56^ reported prolonged persistence of HAdV relative to SOMCPH in facultative pond sludge, influenced by pH, dissolved oxygen, temperature, and conductivity. Effective removal of resistant viral indicators, therefore, requires consideration of viral structure and optimization of WSP operational conditions.

^64–66^. The poor correspondence between bacterial indicator reductions and viral persistence further supports reliance on viral indicators such as crAssphage in risk-based regulation. Geographic variation, WWTP capacity, and methodological differences in sampling and quantification contribute to observed differences in viral concentrations compared with other studies. CrAssphage concentrations in raw sewage were lower than reported in the USA, Spain, Japan, and the UK^64–67^ but comparable to Kongprajug et al.^68^, while effluent concentrations were similar to previous reports^19^.

Epidemiological studies indicate a high prevalence of HAdV and RoV A in Egyptian wastewater^20,69–71^. HAdV is associated with gastrointestinal, respiratory, and urinary infections and serves as a reliable indicator of human fecal contamination^72–74^. Because RoV is not included in the children’s free mandatory vaccination program in Egypt, the burden of RoV gastroenteritis remains high, with a wide diversity of circulating genotypes in the community^75–78^. RoV is also known for its resistance to traditional water and wastewater disinfection methods, particularly chlorine^79,80^. Due to its health risk and resistance to chlorine, the WHO has identified RoV as a reference pathogen in drinking water^81^ Accordingly, the WHO recommends including the RoV vaccine in all national immunization programs, particularly in countries with high child mortality associated with RoV gastroenteritis^82^. Wastewater-based studies in Egypt and elsewhere have consistently reported high RoV detection rates in influent and effluent samples^20,75,83–85^, indicating the limited efficiency of conventional treatment processes in viral removal.

### Microbial interactions and correlations

**Fig. 5:**
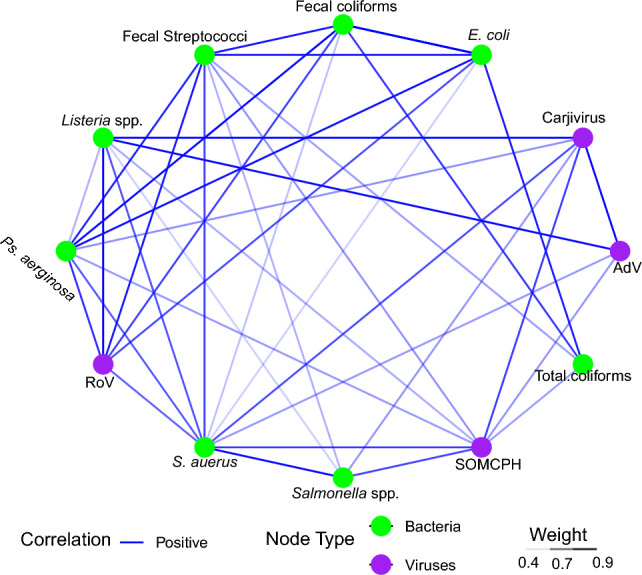
Correlation network between bacteria and viruses based on significant correlations (p < 0.05 and |r| > 0.3). Green nodes represent bacterial parameters, and purple nodes represent viral parameters. Blue edges denote positive correlations. The thickness and transparency of the edges correspond to the strength of the correlation, highlighting key interactions between bacterial and viral communities.

To further investigate microbial interactions, the network visualization revealed strong positive correlations among bacterial indicators, consistent with their shared fecal origin (Fig. 5). TC, FC, and *E. coli* are hierarchically related, with *E. coli* as a subset of fecal coliforms, which in turn are a subset of total coliforms, while fecal streptococci commonly coexist with these markers. These strong associations likely reflect their common source rather than independent interactions.

Moderate to low correlations were observed between viral markers and bacterial indicators. Notably, crAssphage showed a strong correlation with HAdV, supporting its use as a human-specific viral indicator^19,86–89^. Furthermore, crAssphage occurrence in sewage-impacted waters has been associated with an increased risk to human health^90^. In contrast, SOMCPH correlations were weaker, likely reflecting methodological differences between qPCR and cultivation methods and distinct persistence dynamics^91,92^. CrAssphage and HAdV showed moderate correlations with fecal indicators, indicating partial independence from bacterial markers, whereas Salmonella spp., *P. aeruginosa*, and *S. aureus* showed strong associations with *E. coli* (r = 0.93, 0.91, 0.61, respectively; Fig. 5). Variability in correlations, particularly for Listeria spp., underscores the importance of multiple microbial indicators to comprehensively assess fecal contamination and wastewater-associated health risks. Overall, the network analysis emphasizes that no single marker fully captures wastewater contamination, highlighting the complementary roles of bacterial, viral, and pathogen indicators.

### Microbial fate and persistence

In the activated sludge system (WWTP-A), most bacterial indicators and many bacterial pathogens are removed primarily by partitioning to biomass flocs (adsorption/bioflocculation) followed by secondary clarification, and chlorination, which explains the multi-log reductions observed for TC/FC/*E. coli*/FS and the monitored pathogens in this study. However, viruses are smaller and can remain freely suspended or associated with fine particles, making them less dependent on settling and more dependent on inactivation mechanisms; consequently, viral reductions were modest compared with bacterial reductions^93,94^.

In WSP (WWTP-B), microbial removal is driven by a combination of sedimentation, predation, and environmental inactivation (sunlight exposure, elevated temperature, and pond conditions over long retention times). Nevertheless, high suspended solids and algal biomass can shield microbes (particularly viruses) from UV, and pond systems can show more variable performance, consistent with the observed effluent concentrations and the more limited viral log-reductions^34,58^.

Despite significant reductions, treated effluents still contained measurable microbial loads. Indicator bacteria remained at ~3.7–5.0 log₁₀ MPN/100 mL, and the monitored bacterial pathogens were detected at ~2.3–3.0 log₁₀ CFU/100 mL in WWTP-A effluent, with comparable orders of magnitude but greater variability observed in WWTP-B, indicating incomplete removal of potentially health-relevant organisms. For viruses, effluent concentrations in both WWTPs were ~10^4^–10⁶ genome copies/L, with lower and more variable log-reduction found in WWTP-B, a range commonly used in QMRA applications for reuse scenarios^95,96^.

It should be noted that Egyptian Code 501/2015 (Grade D) is intended for restricted reuse applications such as irrigation of trees and non-food crops. Under these conditions, direct dietary exposure is limited; however, public-health relevance remains through non-ingestive pathways, including occupational exposure of workers, dermal contact with reclaimed water, inhalation of aerosols during irrigation, and indirect environmental dissemination. In this context, the persistence of human enteric viruses in treated effluents, even when indicator-based regulatory limits are occasionally met, indicates that residual microbial loads may still pose infection risks under realistic exposure scenarios, particularly in the absence of additional protective measures.

## Conclusions

This study demonstrates clear performance differences between activated sludge and WSP systems in removing organic matter and bacterial contaminants under full-scale conditions. WWTP-A consistently outperformed WWTP-B for COD, BOD, nutrients, and bacterial indicators, reflecting the effectiveness of bioflocculation, adsorption, and clarification processes. In contrast, WWTP-B exhibited lower and more variable performance, influenced by algal activity and environmental conditions.

Despite these differences, a key finding is that both treatment systems failed to adequately remove resistant viral targets, including HAdV, RoV A, and crAssphage. Viral removal remained substantially lower than bacterial removal, even in the better-performing activated sludge system, demonstrating that conventional treatment processes optimized for bacterial and organic matter removal do not reliably control enteric viruses.

Correlation and network analyses further showed that bacterial indicators cannot reliably predict viral occurrence or persistence in treated effluents. Strong associations among bacterial indicators contrasted with weak and inconsistent relationships between bacterial and viral markers, indicating that compliance with bacterial standards does not ensure virological safety.

From a public health perspective, the persistence of viral markers in treated effluents is directly relevant for quantitative microbial risk assessment and wastewater reuse. These findings support the adoption of virus-specific log-reduction targets and the use of crAssphage as a viral process indicator within risk-based wastewater regulation frameworks.

## Supplementary Information

Supplementary Information.

## Data Availability

Data sets generated or analysed during the current study are available upon request.
