# Supplementary material for: Comparative performance of activated sludge and waste stabilization ponds for the removal of pollutants and pathogens in full-scale wastewater treatment plants in Egypt
Source: Sci Rep. 2026 Feb 5;16:5266. doi: 10.1038/s41598-026-35933-4 (PMC12881621; doi:10.1038/s41598-026-35933-4)
Supplement: Supplementary file 1 — Supplementary Information. [file 41598_2026_35933_MOESM1_ESM.pdf]

**Table S1- Egyptian regulations for the treated wastewater reuse, code (501/2015)**

| **Parameters** | **Unit** | **Egyptian ministerial Decree 48/1982** | **Egyptian Code 501/2015 (grade D)** |
|---|---|---|---|
| pH | | 6-9 | - |
| Chemical oxygen demand | mg/l | 80 | - |
| Biological oxygen demand | mg/l | 60 | 350 |
| Total suspended solids | mg/l | 50 | 300 |
| Total Kjeldahl nitrogen | mg/l | - | - |
| Total phosphorous | mg/l | - | - |
| Oil & Grease | mg/l | 10 | - |
| Total Coliform | MPN Index/100 ml | 5000 | - |
| Fecal Coliform | MPN Index/100 ml | - | - |
| *E. coli* | MPN Index/100 ml | - | - |
| *Nematode ova* | Ova/l | <1 egg/l | - |

-Unspecified
